# Scrub Typhus Leading to Acute Liver Failure in a Pregnant Patient

**DOI:** 10.7759/cureus.10191

**Published:** 2020-09-01

**Authors:** Saurabh Gaba, Sanjana Sharma, Nayana Gaba, Monica Gupta

**Affiliations:** 1 General Medicine, Government Medical College and Hospital, Chandigarh, IND; 2 Obstetrics and Gynaecology, Postgraduate Institute of Medical Education and Research, Chandigarh, IND; 3 Obstetrics and Gynaecology, Government Medical College and Hospital, Chandigarh, IND

**Keywords:** scrub typhus, pregnancy, acute liver failure, fulminant liver failure, rickettsia, orientia tsutsugamushi, outcome, multiorgan dysfunction, fetal loss, trombiculid

## Abstract

Scrub typhus is a mite-borne rickettsial infection that presents with fever and a diverse array of complications. Lately, many epidemics have been reported from the Indian subcontinent. Data from these outbreaks suggest that liver injury in scrub typhus is common and reversible. We are reporting the case of a 27-year-old pregnant female who presented with fever, encephalopathy, jaundice and seizure. She had acute liver failure and dead fetus on admission. Despite appropriate antibiotics and supportive treatment, she continued to deteriorate and developed multiorgan dysfunction, leading to her demise.

## Introduction

Scrub typhus is caused by the bacterium *Orientia tsutsugamushi* and transmitted by the bite of chigger larva of trombiculid species. It has recently assumed prominence in India and neighboring countries as numerous outbreaks have been reported [1]. The data collected from these outbreaks have brought to forefront the wide gamut of previously unknown manifestations. The liver injury manifests in the form of jaundice and elevation of the transaminase enzymes. These perturbations are reversible upon successful treatment. We are reporting a case of acute liver failure in a pregnant patient in the setting of scrub typhus. Acute liver failure is defined as development of encephalopathy and coagulopathy (International normalized ratio ≥1.5) after an acute insult (<26 weeks), without any pre-existing liver disease [2].

## Case presentation

**Figure 1:**
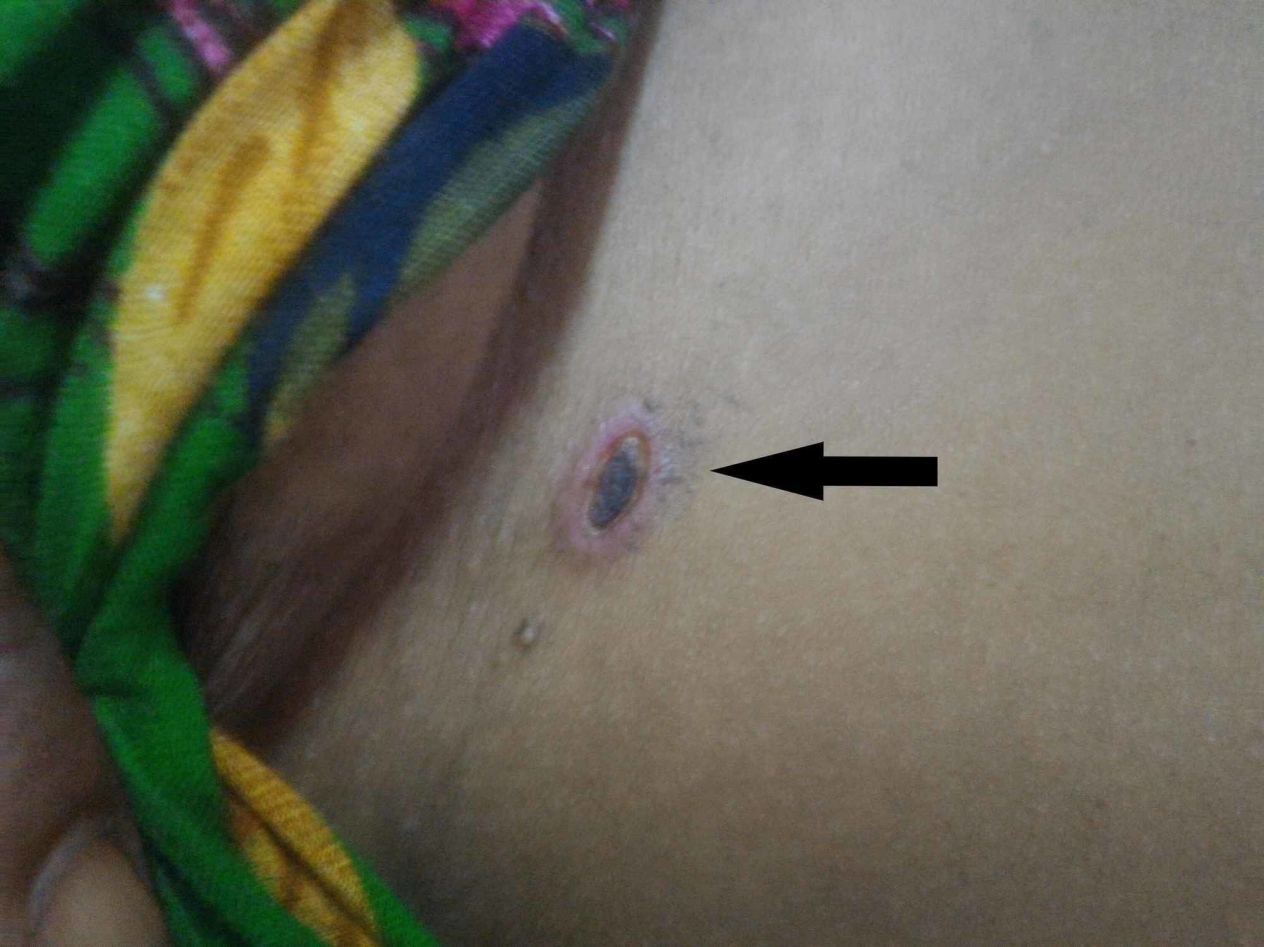
Eschar below right breast. It is present at the site of bite of the vector and visible as a black necrotic scab surrounded by a ring of erythema.

A 27-year-old primigravida at 28 weeks of gestation, residing in a village and involved in agriculture, developed fever followed by jaundice and drowsiness with irrelevant talks three days later. She was brought to the casualty after having a generalized tonic-clonic convulsion. She was somnolent with a Glasgow Coma Score (GCS) of 10/15. Physical examination revealed icterus and pitting pedal edema with an axillary temperature of 102°F, a regular pulse of 105/minute and respiratory rate of 20/minute. Jugular venous pressure was normal. Blood glucose was 53 mg/dL and oxygen saturation by pulse oximetry was 97%. An eschar (Figure 1) was observed below the right breast. There was no lymphadenopathy, rash or other bleeding manifestations. The pupils were reactive and of normal size. She was moving all limbs and meningeal signs were absent.

On respiratory examination, breath sounds were vesicular with no added sounds. Likewise, the cardiovascular examination was unremarkable except for tachycardia. Fetal movements were absent and fetal heart could not be auscultated. Abdominal examination revealed hepatomegaly with the liver palpable 2 cm below the costal margin in the midclavicular line. She had undergone her last antenatal checkup at 23 weeks of gestation, and at that time she had normal blood pressure records with no protein in the spot urine sample, normal thyroid, renal and liver functions, and had tested negative for hepatitis B, hepatitis C, HIV and syphilis. She was only taking iron and calcium supplements. She had never received a blood transfusion, and there was no history of intake of alcohol, recreational drugs and over-the-counter or complementary medicines. The initial blood investigations have been mentioned in Table 1. 

**Table 1:** Initial clinical investigations. ALP, alkaline phosphatase; GGT, gamma glutamyl transferase; AST, aspartate transaminase; ALT, alanine transaminase; PT, prothrombin time; APTT, activated partial thromboplastin time; ESR, erythrocyte sedimentation rate; CRP, C-reactive protein.

Investigation	Value	Normal range
Hemoglobin (g/dL)	10	13-16
Platelets (×10^9^/L)	98	150-400
Total leukocyte count (×10^9^/L)	1.1	4-12
Bilirubin (mg/dL)	11	0.2-1
ALP (U/L)	404	30-150
GGT (U/L)	125	<50
AST (U/L)	1200	10-40
ALT (U/L)	990	10-40
Albumin (gm/dL)	2.8	3.5-5.5
Globulin (gm/dL)	4	2-3.5
PT (seconds)	21	Control-14
APTT (seconds)	50	Control-32
Sodium (mmol/L)	133	135-145
Potassium (mmol/L)	4.1	3.5-5.5
Urea (mg/dL)	58	15-40
Creatinine (mg/dL)	1.3	<1.3
ESR (mm in first hour)	64	<10
CRP (mg/L)	76	<5
Procalcitonin (ng/mL)	1.2	<0.5
Lactate (mmol/L)	2.7	0.5-1

Peripheral blood film did not reveal malarial parasite, and the urine examination did not reveal any proteinuria or red blood cells. Arterial blood gas analysis was suggestive of uncompensated respiratory alkalosis with a pH of 7.49 and partial pressure of carbon dioxide (pCO_2_) of 28 mmHg. Ultrasound of the abdomen revealed mild hepatosplenomegaly and ascites with absent fetal cardiac activity. Venous Doppler was done to rule out hepatic venous outflow obstruction. Non-contrast CT scan of the head displayed diffuse cerebral edema. An electrocardiogram (ECG) showed sinus tachycardia with non-specific ST-T changes, and the initial chest radiograph was normal. The patient was diagnosed to have scrub typhus by detecting IgM antibodies using enzyme-linked immunosorbent assay (ELISA) with an optical density (OD) value of 1.21 (cut-off for positive result was 0.5). The diagnosis of scrub typhus was further confirmed by real-time polymerase chain reaction (PCR). Serologies were negative for hepatitis A, B, C and E, leptospirosis, dengue, chikungunya and malaria, and the blood culture was sterile.

Upon presentation, the patient was intubated and empirical treatment was started with intravenous ceftriaxone (for leptospirosis), artesunate (for complicated malaria) and azithromycin (for scrub typhus). Azithromycin was substituted with doxycycline after fetal death was confirmed ultrasonically. Supportive treatment was given with intravenous fluids, pantoprazole, mannitol, antiepileptic drug levetiracetam and transfusion of fresh frozen plasma. No intervention regarding the dead fetus was agreed upon with the obstetrics team. There was a poor response to therapy, and the patient’s sensorium worsened progressively. There was no recurrence of seizures, and she developed petechiae and hematuria with elevated fibrin degradation products and low plasma fibrinogen, indicating disseminated intravascular coagulation (DIC). Urine output decreased and creatinine rose to 4 mg/dL. She also developed pulmonary infiltrates and hypoxemia. Inotropic support with norepinephrine had to be initiated along with peritoneal dialysis. The patient died due to refractory shock on the fourth day of admission.

## Discussion

Scrub typhus can have a variety of clinical manifestations, and it is not possible to differentiate it from other tropical infections on clinical grounds alone. It can lead to hepatitis, acute kidney injury, meningoencephalitis, DIC, myocarditis, pericarditis and shock [3]. The bacterium undergoes lymphatic and hematogenous dissemination from the site of entry into the body, and the pathogenesis involves endothelial injury and vasculitis. A black necrotic scab, called an eschar, may be present at the site of bite of the vector. It is a very valuable finding; however, it is painless and non-pruritic, and may not be obvious until a thorough inspection is done since it can be present anywhere on the body surface. It has been reported to occur in up to 44% of the cases [4]. The antibiotic of choice for treatment is doxycycline [3]. In pregnant women and children, azithromycin is used. No vaccine is currently available.

In a study on 33 pregnant patients with scrub typhus, 51.5% had intrauterine death, 42.4% had spontaneous abortion and 9.1% had preterm birth [5]. Another study by Kumar et al. concluded that the clinical features and severity of scrub typhus do not differ between pregnant and non-pregnant women [6]. They found hepatic dysfunction in 49.98% of the cases. In a case series of six pregnant patients from north India reported by Meena et al., three had preterm delivery and the other three had fetal loss [7]. Four women recovered swiftly, one required intensive care, and one died. These data are in contrast to an outbreak from another endemic region in south India in which seven pregnant women recovered uneventfully without any obstetric complications except one, who had a premature delivery culminating in baby’s death due to respiratory distress syndrome [8]. In a retrospective analysis of 42 pregnant women with scrub typhus in which 33% had fetal loss, it was found that the risk of fetal loss was highest in the first trimester and the risk decreased with advancing gestational age [9]. Acute liver failure has previously been reported in a pregnant woman with hepatitis E virus and scrub typhus co-infection [10]. It has also been reported in neonates with scrub typhus [11]. Histopathologic examination of the liver on autopsy of a patient with acute liver failure secondary to scrub typhus revealed necrosis of hepatocytes, inflammatory infiltrate in hepatic capsule and fibrin thrombi in the sinusoids [12]. These findings were suggestive of DIC as the possible mechanism.

The burden of infectious diseases is high in India. Tropical infections like dengue, malaria, leptospirosis, scrub typhus and enteric fever can present with an undifferentiated syndrome of fever associated with certain complications, which can lead to diagnostic difficulty. Thrombocytopenia, hepatitis and encephalopathy can occur in either of these diseases [13]. Deepak and Patel have described a case series of 28 patients of acute liver failure in India [13]. Two patients had scrub typhus, and the other cases were ascribed to viral hepatitis, malaria, dengue, leptospirosis, amebic liver abscess, endemic typhus and typhoid fever. The patient under consideration in our report did not have the characteristic macular rash, arthralgia and headache, which are seen in dengue [14]. Enteric fever was unlikely since there was no history of abdominal pain, diarrhea or constipation, and encephalopathy typically occurs in the third week of illness [15]. Viral hepatitis was a strong possibility considering the marked elevation of hepatic transaminases accompanying the fever, and acute liver failure is known to occur in acute hepatitis A, B and E [16]. It was also crucial to rule out leptospirosis since hepatic dysfunction is considered to be its hallmark and the patient lived in a rural area with farms [17]. Non-infectious causes worthy of consideration in this case were pre-eclampsia, eclampsia and acute fatty liver of pregnancy. Her normal antenatal blood pressure records, absence of proteinuria, and acute presentation made hypertensive disorders implausible [18]. Acute fatty liver of pregnancy presents with non-specific signs and symptoms, such as nausea, vomiting, fatigue and anorexia, leading eventually to liver failure. It is unusual for it to present before 30 weeks of gestation [19]. The common causes of acute liver failure have been summarized in Table 2 [20].

**Table 2:** Common causes of acute liver failure. HELLP: hemolysis, elevated liver enzymes, low platelets

Infections	Drugs	Toxins	Pregnancy-specific	Others
Hepatitis B, hepatitis E, herpes simplex, Epstein-Barr virus, Cytomegalovirus	Acetaminophen, allopurinol, efavirenz, amiodarone, halothane, pyrazinamide, valproate	Carbon tetrachloride, Amanita phalloides mushroom	Acute fatty liver of pregnancy, pre-eclampsia, eclampsia, HELLP syndrome	Wilson’s disease, sepsis, Budd-Chiari syndrome

## Conclusions

This report describes the case of a 27-year-old pregnant female in third trimester who had fetal loss and died due to acute liver failure with multiorgan dysfunction in the setting of scrub typhus. Literature review has revealed that although liver dysfunction is common in scrub typhus, acute liver failure is a very rare entity, and the risk of fetal loss or premature delivery is high in pregnant women.
